# LiSEQ – whole-genome sequencing of a cross-sectional survey of *Listeria monocytogenes* in ready-to-eat foods and human clinical cases in Europe

**DOI:** 10.1099/mgen.0.000257

**Published:** 2019-02-18

**Authors:** Anaïs Painset, Jonas T. Björkman, Kristoffer Kiil, Laurent Guillier, Jean-François Mariet, Benjamin Félix, Corinne Amar, Ovidiu Rotariu, Sophie Roussel, Francisco Perez-Reche, Sylvain Brisse, Alexandra Moura, Marc Lecuit, Ken Forbes, Norval Strachan, Kathie Grant, Eva Møller-Nielsen, Timothy J. Dallman

**Affiliations:** ^1^​ Public Health England Gastrointestinal Bacteria Reference Unit, 61 Colindale Avenue, London, NW9 5EQ, UK; ^2^​ National Institute for Health Research Health Protection Research Unit (NIHR HPRU) in Gastrointestinal Infections at University of Liverpool, Liverpool, UK; ^3^​ Statens Serum Institut, 5 Artillerivej, DK-2300, Copenhagen S, Denmark; ^4^​ Maisons-Alfort Laboratory for Food Safety, Salmonella and Listeria Unit, University of Paris-Est, French Agency for Food, Environmental and Occupational Health & Safety (ANSES), Maisons-Alfort, France; ^5^​ School of Biological Sciences, The University of Aberdeen, Cruickshank Building. St Machar Drive, Aberdeen, Scotland, AB24 3UU, UK; ^6^​ Institute of Complex Systems and Mathematical Biology, SUPA, School of Natural and Computing Sciences, University of Aberdeen, Aberdeen, Scotland, UK; ^7^​ Institut Pasteur, Paris, France; ^8^​ School of Medicine and Dentistry, The University of Aberdeen, Foresterhill, Aberdeen, Scotland, AB25 2ZD, UK

**Keywords:** *Listeria monocytogenes*, whole-genome sequencing, genetic diversity, phylogeny, food, human

## Abstract

We present the LiSEQ (Listeria SEQuencing) project, funded by the European Food Safety Agency (EFSA) to compare *Listeria monocytogenes* isolates collected in the European Union from ready-to-eat foods, compartments along the food chain (e.g. food-producing animals, food-processing environments) and humans. In this article, we report the molecular characterization of a selection of this data set employing whole-genome sequencing analysis. We present an overview of the strain diversity observed in different sampled sources, and characterize the isolates based on their virulence and resistance profile. We integrate into our analysis the global *L. monocytogenes* genome collection described by Moura and colleagues in 2016 to assess the representativeness of the LiSEQ collection in the context of known *L. monocytogenes* strain diversity.

## Data Summary

All fastq reads from this study have been deposited in the National Center for Biotechnology Information short-read archive (SRA) under the BioProject PRJNA475189.

**Significance as a BioResource to the community**

The LiSEQ (Listeria SEQuencing) genome collection represents a valuable resource of *Listeria monocytogenes* for further study. It provides a framework to answer questions on genetic diversity amongst different sources assayed in this strain collection, as well as to explore possible epidemiological links between isolates from across Europe.

## Introduction

*Listeria monocytogenes* is an opportunistic pathogen that causes a range of illnesses from mild febrile gastroenteritis to more severe invasive infections, including bacteraemia and meningitis (listeriosis) [1]. Whilst listeriosis is a relatively rare disease, it has a high fatality rate of 20–30 % and, therefore, the burden of the disease is high [2]. Some populations have an increased susceptibility, including the elderly, immunosuppressed patients, pregnant women, their foetuses and neonates [1]. The majority of cases appear to be sporadic, although outbreaks are not uncommon. In the European Union (EU) in 2016, a total of 2536 confirmed human cases were reported by 28 member states, corresponding to an EU notification rate of 0.47 cases per 100 000 population. The highest notification rates were observed in Finland, Belgium, Germany, Slovenia and Denmark, with 1.22, 0.92, 0.85, 0.73 and 0.70 cases per 100 000 population, respectively [3].

Cases of listeriosis are frequently associated with the consumption of contaminated ready-to-eat (RTE) food products, with meat and fish products and soft and semi-soft cheeses often identified as vehicles of infection [2]. *L. monocytogenes* can be found in both raw foods and in processed foods that are contaminated during and/or after processing. *L. monocytogenes* can survive and replicate at low temperatures (with a minimal growth temperature of −2 °C) and has the capacity to persist in food-processing environments, sometimes for years [4]. Contamination of food-processing environments is often the route by which RTE food becomes contaminated [5] and those foods with a relatively long shelf life are of particular public-health concern [6]. Identifying potential food vehicles and understanding how foods become contaminated is paramount to developing and implementing effective control and preventative measures, and the typing of *L. monocytogenes* isolates plays a crucial role in such investigations [7–9].

An EU-wide baseline survey (BLS) was conducted in 2010 and 2011 to estimate the prevalence of *L. monocytogenes* contamination in three RTE food categories at retail in accordance with EU decision 2010/678/EU: packaged (not frozen) smoked or gravad fish (3053 samples), packaged heat-treated meat products (3530 samples) and soft or semi-soft cheeses (3452 samples). The prevalence estimates were published in 2013 [10]. The percentage of fish in the EU contaminated with *L. monocytogenes* at the time of sampling was 10.4 % and at the end of shelf-life 10.3 %, whilst the level for contaminated meat and cheese samples at the end of shelf-life was 2.07 and 0.47 %, respectively. In the EU, the proportion of smoked or gravad fish samples with a *L. monocytogenes* count exceeding the level of 100 c.f.u. g^−1^ was 1.7 % at the end of shelf-life. For meat products, this proportion was 0.43 %, whilst for the cheese it was 0.06 %.

Several phenotypic and genotypic methods have been used worldwide for typing *L. monocytogenes*. Traditionally, serotyping, based on the agglutination of somatic (O) and flagellar (H) antigens, classifying *L. monocytogenes* into at least 13 serotypes, has been the first level of subtype discrimination [11]. However, as only three serotypes cause over 95 % of invasive infection, molecular typing methods are employed for greater strain discrimination with PFGE being, until recently, the gold-standard method for *L. monocytogenes* [12].

Multilocus sequence typing (MLST) has been used to study and describe the population structure and phylogeny of many bacterial pathogens, and has shown that *L. monocytogenes* forms a structured population consisting of four divergent lineages (I– IV) [11, 13]. Each lineage comprises specific serotypes: with lineage I containing serotypes 1/2b, 3b, 4b, 4e and 7; lineage II, serotypes 1/2a, 1/2 c, 3a and 3 c; lineage III, serotypes 4b, 1/2a, 4a and 4 c; and lineage IV, 4a and 4 c. The genetic lineages have different, although at times overlapping, genetic, phenotypic and epidemiological characteristics with the majority of human illness caused by strains in lineages I and II [11].

With the advent of whole-genome sequencing (WGS) technologies, entire bacterial genomes are now readily available for analysis affording the highest level of strain discrimination, the ability to infer phylogenetic relationships and access to a wealth of additional information such as virulence and resistance markers. A recently developed core genome MLST scheme has been described for *L. monocytogenes* by Moura and colleagues [7] encompassing 1748 loci, which has been used to describe the global population structure of the species. Furthermore, WGS has been used in several national studies for outbreak detection and investigations, e.g. in Austria [14], Australia [15], the USA [16], Denmark [17] and France [18]. The improvements in strain resolution obtained with WGS analyses provided robust genetic evidence for linking cases and more accurate case definitions than PFGE, enabling cases to be ruled in or out of outbreaks.

We present results derived from a study funded by the European Food Safety Agency (EFSA) to compare *L. monocytogenes* isolates collected in the EU from RTE foods, compartments along the food chain (e.g. food-producing animals, food-processing environments) and humans. In this article, we report the molecular characterization of a selection of *L. monocytogenes* isolates from the above sources, and human clinical cases employing WGS analysis.

## Methods

### Strain selection

A total of 1143 *L. monocytogenes* isolates were selected to be part of the LiSEQ (Listeria SEQuencing) study. These encompassed and included those from the EU-wide RTE BLS [10] and were collected from: different compartments of the food-production chain (*n*=200); sporadic clinical cases (*n*=262) that were temporally and geographically matched to the RTE BLS (*n*=353); and isolates associated with outbreaks (*n*=105). The selected BLS isolates consisted of 353 strains originating from 22 member states and 1 non-member state with 297, 49 and 7 strains isolated, respectively, from RTE fishery products, meat products, and soft and semi-soft cheeses. To compensate for the excess of BLS isolates from RTE fish products, additional isolates (*n*=223) from RTE meat products and cheeses, collected during the years 2010–2011, were obtained from eight different EU member states to ensure a more equal distribution of isolates across each of the three RTE food categories. Clinical isolates from assumed sporadic human cases collected during the BLS period 2010–2011 were also included. Selection priority was according to availability of the isolate and the complementary typing data and country disease incidence. Additionally, isolates from raw food sampled at fish, meat and milk-product production sites, as well as environmental isolates from these sites, were also included. Isolates associated with nine retrospective outbreaks, including those from human cases and, where applicable, the confirmed food source, were selected, representing outbreaks with different sources and occurring in different geographical regions. A summary of the final set of strains included in the project is given in Table 1 and a complete table of meta data is provided in Table S1 (available with the online version of this article).

**Table 1.** Summary of the strains included in the LiSEQ study

**Country**	**BLS**	**Other foods**	**Food-production chain**	**Clinical, sporadic**	**Outbreak**	**Total**
A	7	29		35		71
B	4	28	68	31	43	174
C	35	83	32	35	25	210
D	4			20		24
E	6					6
F	15			8		23
G	4	4				8
H	5					5
J	10					10
K	14					14
L	54					54
M	2					2
N	9					9
P	3	4				7
Q	33		100	23		156
R	4					4
S	4					4
T	4			20	5	29
U	62					62
V	6	28				34
W	7			15		22
X	38	34		35	32	139
Y	8			20		28
Z	15	13		20		48
**Total**	**353**	**223**	**200**	**262**	**105**	**1143**

To assess the representativeness of the genetic diversity afforded by the isolates in this study, the LiSEQ results were placed into a global context via comparison with the *L. monocytogenes* collection of 1696 genomes from Moura and colleagues [7], which represent a larger geographically distributed data set.

### Sequencing and bioinformatics analysis

Bacterial isolate growth was harvested into a 96-well processing plate and treated with lysozyme at 37 °C for 1 h, followed by proteinase K overnight at 56 °C with gentle shaking. Lysates were heated to 95–100 °C for 10 min to ensure inactivation of any non-lysed bacterial cells. Samples were then treated with ribonuclease A for 15 min at 37 °C, centrifuged and the supernatants transferred to an automated nucleic acid extraction platform, Qiagen’s QiaSymphony. The yield and purity of extracted DNA was assessed using the Life Technologies Quant-iT high sensitivity 96-well assay and the GloMax Multi+Detection and LabChip DX systems. DNA was diluted to 10–30 ng µl^−1^.

Paired-end libraries were generated using the Illumina Nextera XT sample preparation kit. Assessment of fragment sizes was performed on the Perkin Elmer LabChip GX after fragmentation and clean-up. After normalization, samples were pooled and library quantification was performed using the KAPA library quantification kit for Illumina sequencing, on an ABI ViiA7 system. Paired-end sequencing was performed on the Illumina HiSeq 2500 instrument using the TruSeq Rapid SBS kit (200 cycle) and TruSeq paired-end rapid cluster kit. The following cycle parameters were used for sequencing: read 1 : 101, index read 1 : 8, index read 2 : 8 and read 2 : 101. rta version 1.17.21.3 was used for generation of base call files. fastq creation and de-multiplexing via casava was performed on a dedicated high performance cluster (HPC). fastq reads were quality trimmed using Trimmomatic [19] with bases removed from the trailing end that fell below a phred score of 30. If the read length post-trimming was less than 50, the read and its pair were discarded. If the post-trimmed yield was less than 150 megabases, the sample was discarded. A kmer (a short string of DNA of length k) based approach was used (https://github.com/phe-bioinformatics/kmerid) to confirm the identity of the sample and to ensure the sequence was free from contamination. If any non-*Listeria* kmers were identified in the fastq reads, the sample was discarded. All fastq reads from this study can be found in the National Center for Biotechnology Information short-read archive under BioProject PRJNA475189.

The MLST sequence type (ST) as defined by the Pasteur scheme [13] was extracted from each sequence using most (https://github.com/phe-bioinformatics/MOST) [20] and assigned a clonal complex (CC) in accordance with the Institut Pasteur international MLST database for *L. monocytogenes* designation (http://bigsdb.pasteur.fr/listeria). One preselected isolate was found to be *Listeria innocua*; thus, it was excluded from further analysis.

Short reads were assembled using Spades assembly (version 3.5.0) run with Kmer 21, 33, 55, 77, 83, and the ‘only-assembler’ option [21]. Core genome SNP phylogenies were constructed on the obtained assemblies using Parsnp [22].

Resistance to tetracycline, penicillin, quaternary ammonium sanitizers and antiseptics (such as benzalkonium chloride) were assayed *in silico* from the genomes of the isolates in this study. Tetracycline resistance was inferred by the presence of *tetM* and *tetS*, and penicillin resistance by the presence of *penA.* Resistance to quaternary ammonium sanitizers and antiseptics was inferred by the presence of the *bcrABC* locus, the *Tn6188_qac* transposon, and/or the efflux pumps *emrE* [23] and *qacA* [24]. For detection of gene presence, ‘paired-end’ reads of each strain were mapped against the reference gene sequences using Bowtie2 v.2.2.5. [25]. The resulting alignment sam files were then converted into bam files and sorted by using SAMtools [26]. Genes were defined as detected if they covered greater than 80 % of the query sequence with greater than 80 % nucleotide identity. Genes with coverage less than 100 % were also classified as truncated.

A comprehensive set of 115 genes identified as putative or confirmed virulence factors were used as described in two previous studies [27; 28]. The gene sequences were extracted from *L. monocytogenes* EGD-e (accession no. NC_003210.1) except for the *Listeria* pathogenicity island (LIPI)-3/LIPI-4 cluster of genes, which was extracted from *L. monocytogenes* F2365. Genes were detected as above.

## Results

### Population structure

**Fig. 1.**
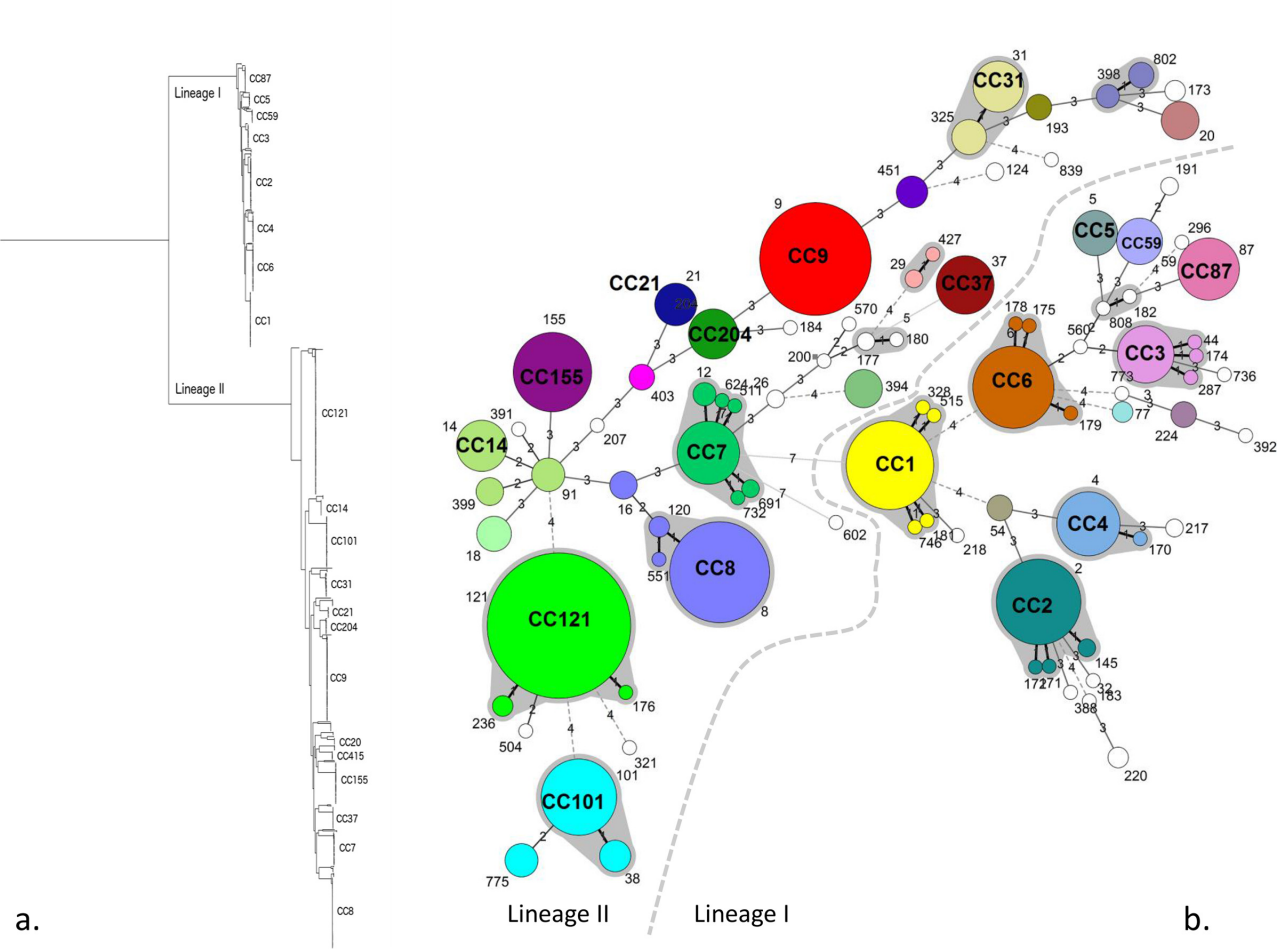
(a) Core genome SNP maximum-likelihood phylogeny of *L. monocytogenes* genome sequences with the clades annotated by 7 loci MLST CC. (b) Minimum spanning tree of the isolates included in this study as described by 7 locus MLST. Each circle represents a single ST that is numbered on the tree. Major CCs defined by single locus variants are shaded in grey. The number of loci that differ between STs is labelled on the branches.

Of the 1143 isolates sequenced, 42 different CCs and 13 singleton STs (unassigned CCs) were identified. One isolate was *L. innocua*; thus, it was excluded from further analysis. One isolate could not be assigned to any ST or CC due to an incomplete MLST profile. Ten CCs accounted for 70 % of the isolates (Table 2). The MLST population structure of the isolates in this study is further described as a minimum spanning tree (Fig. 1b). All isolates in the study clustered in either lineage I or II and the population structure based on whole-genome SNPs is displayed in the phylogenetic tree in Fig. 1(a). From the phylogenetic analysis, it can be seen that there is a clear delineation between lineages and the MLST CCs within lineages.

**Table 2.** The CCs identified and the number of isolates by isolation context and listed in the strain selection information Minor CCs (i.e. CCs with less than 10 isolates) included CC398, CC11, CC193, CC224, CC403, CC54, CC177, CC19, CC220, CC29, CC77, CC217, CC26, CC379, CC207, CC218, CC388, CC475, CC88, CC89, ST184, ST200, ST32, ST382, ST392, ST560, ST570, ST602, ST736, ST773 and ST839 (ordered according to occurrence).

**CC**	**Lineage**	**RTE food**	**Food-chain processing**	**Clinical, sporadic**	**Outbreak related**	**Total**
CC121	II	144	37	6	0	187
CC9	II	81	15	14	0	110
CC8	II	69	5	24	0	98
CC1	I	10	4	50	8	72
CC2	I	19	29	20	0	68
CC101	II	10	41	16	0	67
CC6	I	30	3	28	0	61
CC155	II	32	1	8	13	54
CC7	II	16	4	16	8	44
CC14	II	13	2	9	13	37
CC4	I	1	1	10	24	36
CC87	I	10	0	4	19	33
CC31	II	24	7	1	0	32
CC3	I	18	7	6	0	31
CC37	II	9	15	5	0	29
CC204	II	17	3	1	0	21
CC59	I	10	0	4	4	18
CC5	I	7	6	4	0	17
CC21	II	13	0	2	0	15
CC20	II	8	2	2	0	12
CC415	II	0	2	0	9	11
CC18	II	0	6	4	0	10
Minor CCs	LI=32 LII=47	35	9	28	7	79
**Total**		**576**	199	**262**	**105**	**1142**

There was an uneven distribution in terms of origin of isolates (food, food-processing environment and clinical) between the two lineages (Chi squared test, *P* value 5×10^−29^). Across the CCs, within each lineage there was also an uneven distribution of food, food-processing environment and clinical isolates (lineage I, Chi squared test, *P* value 8.55×10^−24^; lineage II, Chi squared test, *P* value 1.95×10^−30^).

**Fig. 2.**
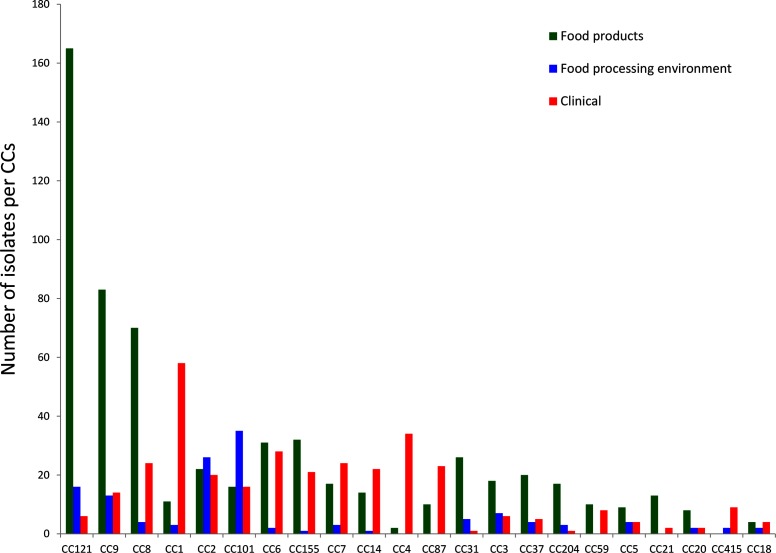
Distribution of CCs in RTE food and from human clinical infections.

**Fig. 3.**
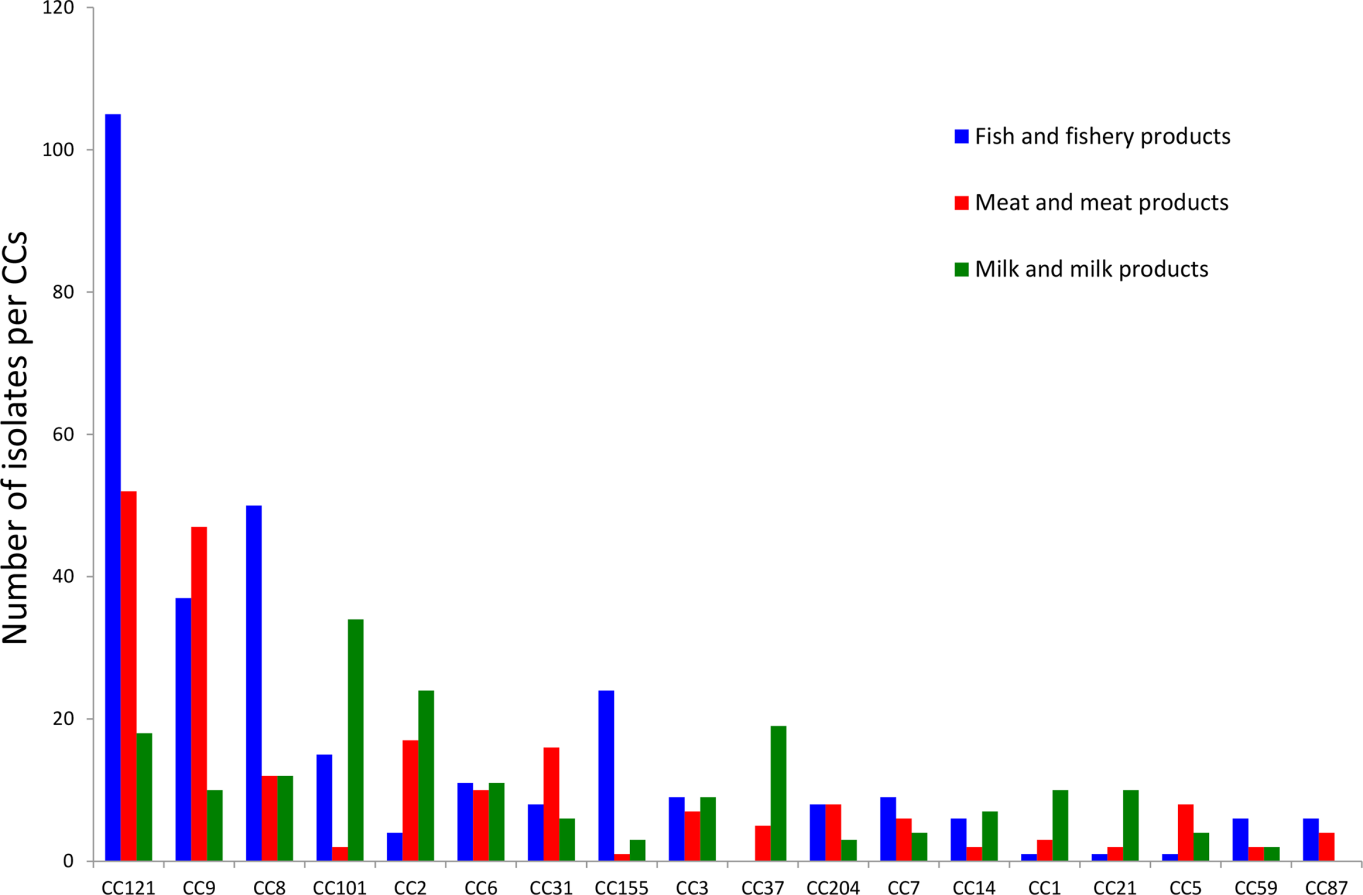
Distribution of CCs from the three major food-product categories, including isolates from food-processing environments.

A total of 51 % of the isolates in lineage I were from humans, compared to only 19 % of the isolates from lineage II. The proportion of food isolates in lineage I was 37% and in lineage II it was 69 %. Across the sampled population, some CCs were significantly enriched with food isolates (e.g. CC121) and others were more associated with human cases (CC1, CC4) (Fig. 2). Some CCs were more representative of a food category, for example CC121 for fish/fishery products and CC101 for milk/milk products (Fig. 3).

**Fig. 4.**
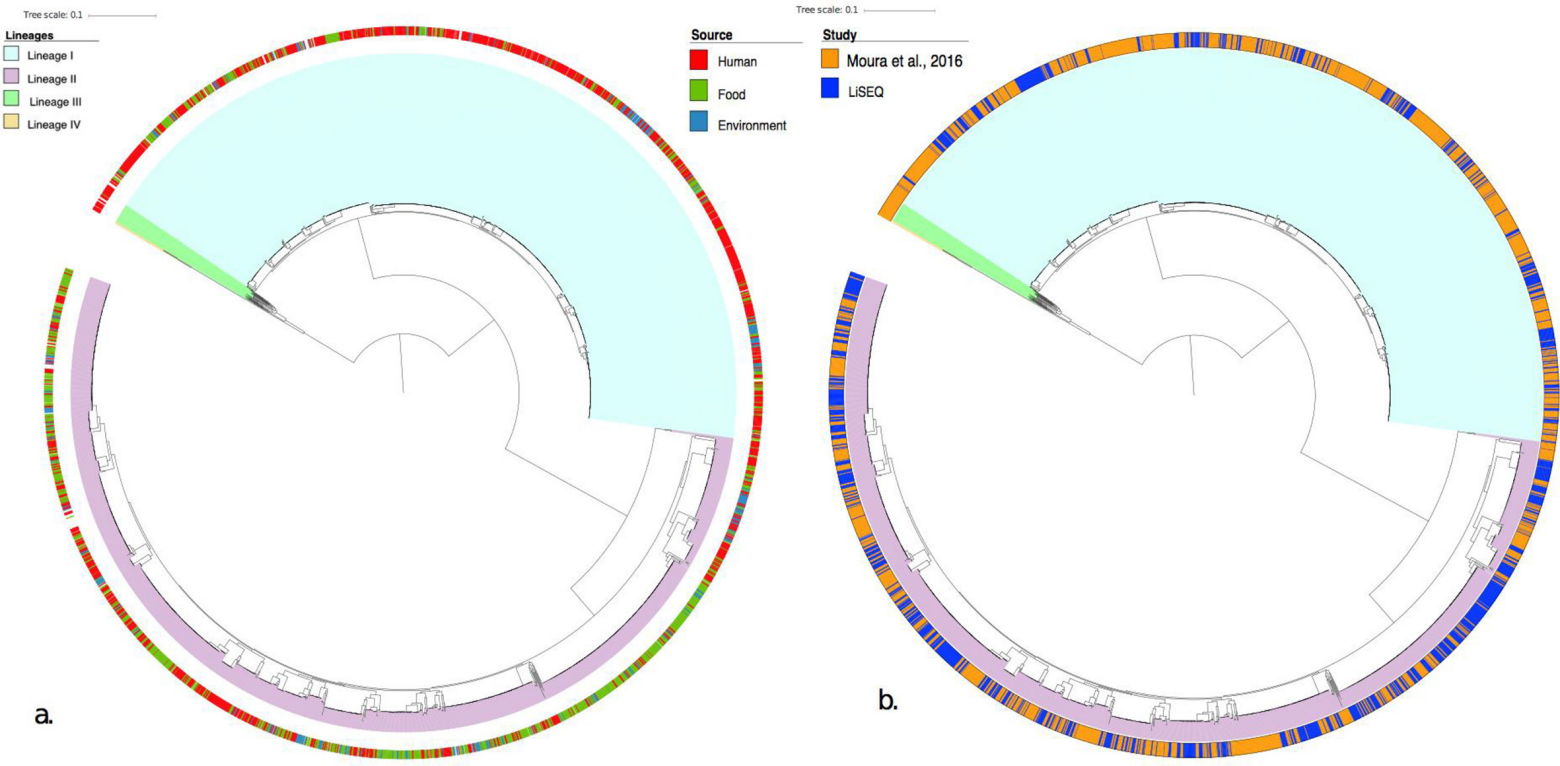
Core SNP tree built with Parsnp showing lineages for *L. monocytogenes*. (a) The external ring shows the source of isolates. (b) The external ring describes the study origin of the isolates: orange, from the study by Moura *et al*. [7]; and blue, from LiSEQ.

To assess how the sampled genetic diversity in this study corresponded to that described in a global context, the 1696 genomes from the study by Moura *et al*. [7] were integrated into the analysis. Fig. 4 shows a core SNP phylogeny of the combined LiSEQ and global data set. The LiSEQ isolates cluster within the global diversity and all major CCs are represented in the study data. A similar distribution of food and clinical isolates are also observed in the data set, with a predominance of clinical isolates found within lineage I and an excess of food isolates found within lineage II (Figs S1 and S2).

### Resistance and virulence

Table 3 shows the percentage of strains in the study collection harbouring the assayed resistance genes. The resistance profile for each strain is included in Table S2. Less than 1 % of isolates harboured tetracycline-resistance genes (*tetM)* with no detection of *tetS*. Benzalkonium chloride-resistance genes were found with 18.5 % of isolates carrying the *Tn6188_qac* transposon and approximately 5 % of isolates carrying *bcrABC* loci. Less than 1 % of isolates harboured the efflux proteins *emrE* and *qacA*, whilst the efflux protein *qacE* was found in 18.3 % of isolates and generally found in conjunction with *Tn6188_qac*. No isolates showed the presence of *penA*, which is involved in resistance to penicillin.

**Table 3.** Percentage of isolates in the study harbouring the assayed resistance genes

**Gene**	Moura et al. [7**18]** **(% detection)**	**LiSEQ** **(% detection)**
*tetM*	0.3	0.6
*tetS*	0	0
*bcrA*	4.6	4.5
*bcrB*	4.5	4.5
*bcrC*	4.5	4.4
*emrE*	0.8	0.3
*qacA*	0.2	0.5
*qacE*	14.9	18.7
*Tn6188_qac*	15.0	18.9
*penA*	0	0

The proportion of resistance determinants observed in the LiSEQ data displays a high correlation with the Moura *et al*. [7] data set. The highest percentage difference was observed with the gene *qacE* and the transposon *Tn6188_qac* efflux mediated benzalkonium chloride resistance, which is approximately 3 % higher in the LiSEQ data set.

**Fig. 5.**
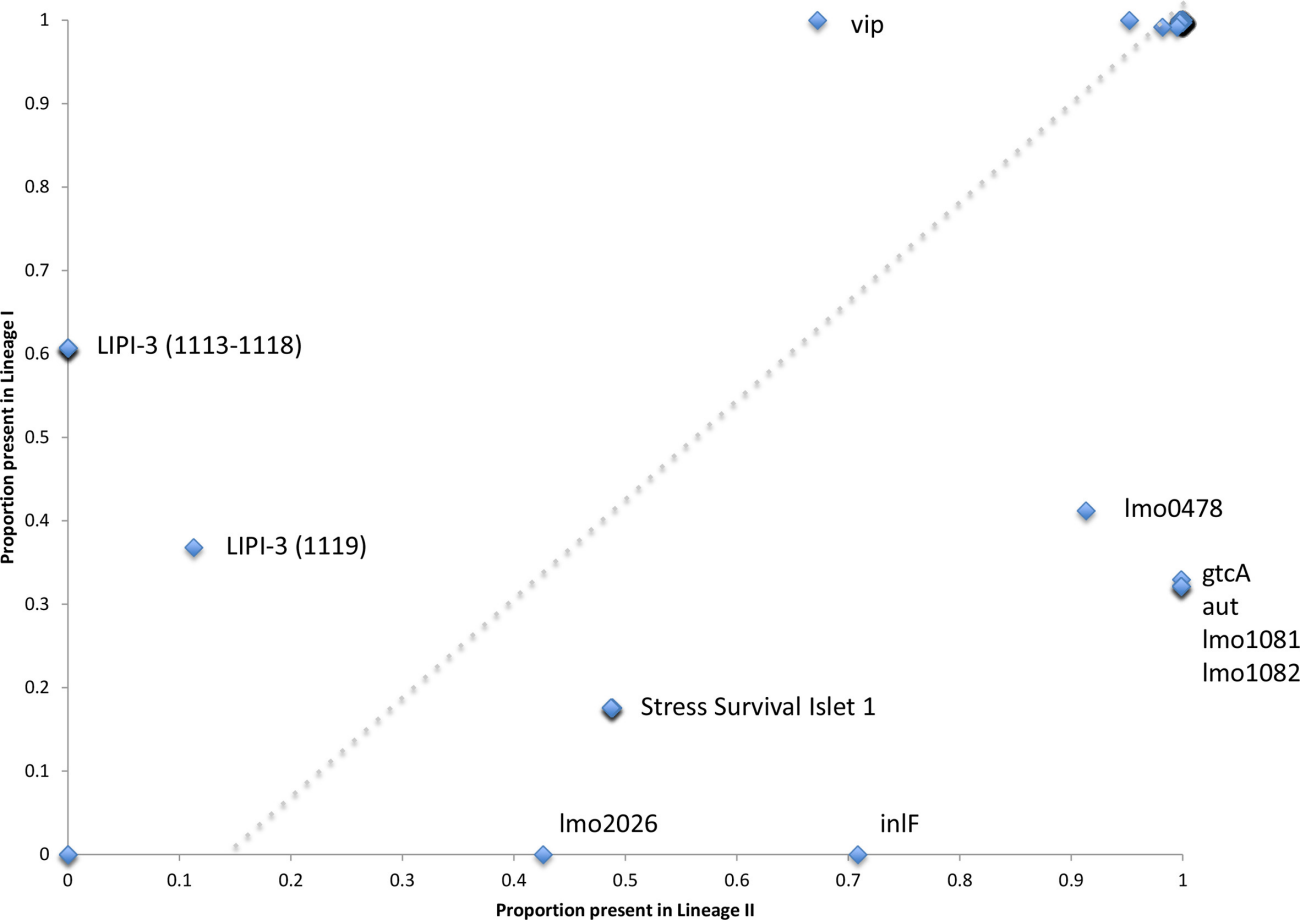
Scatter plot showing the proportion of each of the 115 putative virulence markers found in lineage I or lineage II (only significant results have been labelled).

**Fig. 6.**
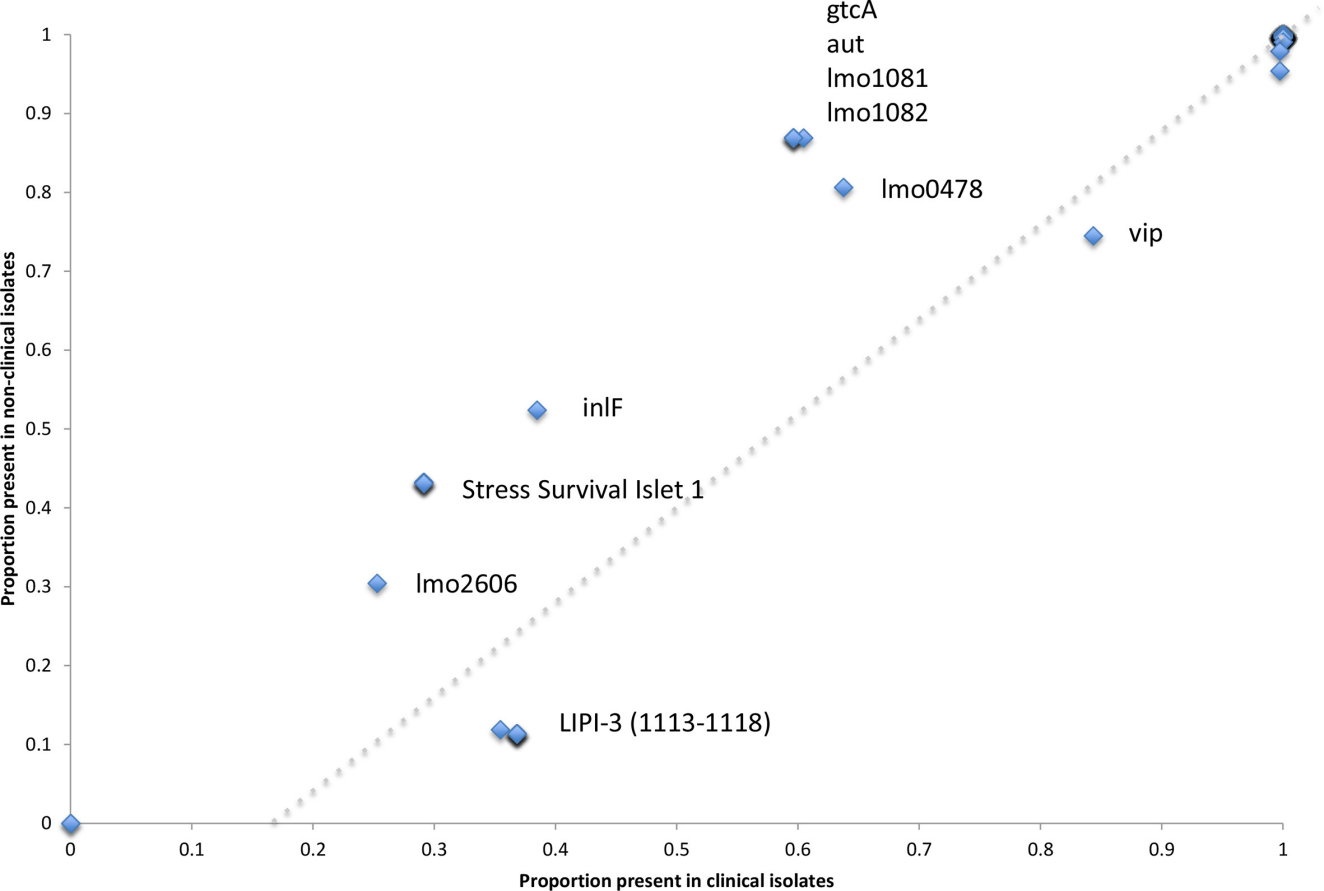
Scatter plot showing the proportion of each of the 115 putative virulence markers found in clinical or non-clinical isolates (only significant results have been labelled).

Table S2 shows the presence and absence of 115 putative virulence markers across the strain collection. Of the 115 markers, 2 were absent across all isolates, conversely 92 markers were present in greater than 95 % of isolates. Fig. 5 shows for each virulence marker the proportion present in linage I and lineage II isolates, and Fig. 6 shows for each virulence marker the proportion present in clinical and non-clinical isolates.

In total, 21 putative virulence markers had significant variability in their detection across the strain collection. As described by Maury *et al.* [28], the *Listeria* pathogenicity island 3 (LIPI-3) was found in 60 % of isolates from lineage I (ubiquitous in CC1, CC3, CC4 and CC6), but completely absent in lineage II isolates. LIPI-3 loci 1119 (*IIsP* gene *LMOf2365_1119*) showed a different presence and absence profile to the other LIPI-3 alleles, being found in a minority of lineage II isolates (12/187 CC121, 11/54 CC155, 14/98 CC8 and 11/110 CC9) in the absence of the other LIPI-3 loci. Conversely, in lineage I some isolates do not possess LIPI-3 loci 1119 (*IIsP* gene) and have an otherwise intact LIPI-3. The *Listeria* pathogenicity island 4 (LIPI-4) was found in only 81 strains (0.07 % of the data set). It was detected in all the CC4 isolates that were tested, in agreement with the findings of Maury *et al* [28], and also in all the CC87 isolates that were investigated.

The known virulence surface protein Vip [29] was found across all isolates in lineage I, but only in 70 % of lineage II isolates (absent in CC204, CC21, CC31 and CC37, and 1/43 of CC7 isolates and 3/98 CC8 isolates). Several putative virulence factors were found in a greater proportion in lineage II isolates compared to lineage I isolates. These included the internalins *lmo2026* (absent in lineage I and ubiquitous in CC155, CC18, CC20, CC204, CC21, CC37, CC415, CC7 and CC9 lineage II isolates) and *inlF* (absent in lineage I and only absent in CC121 and CC14 of lineage II), which have previously been shown to be detected variably in different serotypes [30]. The five gene locus termed the stress survival islet (SSI-1) [31, 32], which has previously been associated with growth of *L. monocytogenes* under sub-optimal conditions, contributing to survival of certain strains in food environments, was over-represented in lineage II isolates. However, when we consider the number of CCs, this association is less clear. SSI-1 is present in CC3 and CC5 of lineage I, and conversely absent in lineage II CCs 101, 121, 14, 20, 21, 415 and 7.

Ubiquitous amongst lineage II isolates was the *rmlACBD* l-rhamnose biosynthesis loci (*lmo1081* and *lmo182*) [33] and *gtcA* [34], both of which are involved in *L. monocytogenes* cell wall teichoic acid production. The former involved in providing protection against the activity of antimicrobial peptides and the later in teichoic acid glycosylation. The autolysin *aut* (*lmo1076*), which has a proposed role in entry of *L. monocytogenes* into non-phagocytic mammalian cells [35], was found across all lineage II isolates, but only in CC3, CC5, CC59 and CC87 of lineage I; however, the shorter variant *LMOF2365_RS00075* was found across all lineage I isolates. Finally, the surface adhesion *lapB* required for entry into mammalian cells is present across all lineages, but absent in all isolates of CC31.

Loss of function through partial gene deletion or miss-sense mutations is also known to be important in virulence attenuation. To explore this, genes with less than 100 % coverage of the query sequence were designated as truncated (see Table S2). Several genes had a loss of function truncation in lineage II but were found intact in lineage I, these included the already described *inlA* deletions [28], as well as *lmo0257*, the terminal SSI loci *lmo0478*, the autolysin gene *ami* and the actin-assembly inducing protein precursor gene *actA*. Conversely several genes were disrupted in lineage I, but intact in lineage II isolates. These included the internalins *inlH*, *inlJ*, *lmo1290*, the stress protein *clpB* and the flagellar motor switch protein *lmo0698*.

## Discussion

The main objective of this study was to compare *L. monocytogenes* isolates collected in the EU from RTE foods, compartments along the food chain and from human cases, and highlights the value of revisiting well-structured surveys. A total of 1142 *L. monocytogenes* isolates were analysed, including 333 human clinical isolates and 809 isolates from the food chain.

Phylogenetic analysis showed a clear delineation between *L. monocytogenes* lineages and between CCs within lineages. The association of isolate type was unevenly distributed across the genetic diversity, with CCs within lineage I strongly associated with clinical cases and lineage II strongly associated with isolates from food. The diversity and distribution observed in this study were consistent with those previously described in a globally representative data set [28, 36–38].

As well as affording high-resolution typing and phylogenetic context, WGS provides immediate access to a wealth of additional data. Antimicrobial resistance in *Listeria* sp. has been studied in various food, environmental and clinical settings [39, 40, 41, 42]. *L. monocytogenes* has generally been shown to be more susceptible to antimicrobial agents than other species in the genus, such as *L. innocua* [31]. In this study, we found remarkable low-prevalence genes encoding resistances to tetracycline (<0.1 %) and penicillin (1 %). Genes conferring resistance to detergents and antiseptics via efflux activity were detected at a prevalence approaching 20 %. Whilst it is encouraging that the isolates in this study show potentially low levels of antimicrobial resistance, it is important to remain vigilant for emerging resistance. WGS allows antimicrobial-resistance monitoring to be done at no additional cost if WGS is part of routine microbial surveillance and, therefore, allows this potential threat to be monitored going forward.

WGS data were also assessed for the presence of 115 putative markers of virulence. More than 80 % of markers were present in more than 95 % of the isolates suggesting that most putative markers described in the literature are ubiquitous across *L. monocytogenes* lineages I and II. The majority of markers not present in all isolates were over-represented in food and/or lineage II isolates, with markers associated with stress survival or cell wall modification being particularly enriched. Conversely, the recently discovered *Listeria* pathogenicity island 3 and the surface protein VIP were more likely to be found in clinical and/or lineage I isolates. Although most virulence markers were present in all strains, it is not known whether the genes are expressed. Further work is needed, including the determination of truncation and non-sense mutations that have been shown to be associated with changes in virulence in particular that associated with the internalin genes [28]. Several truncations were identified in virulence genes across the data set, with some having an increased propensity for truncation dependent on lineage.

The WGS data generated represents a valuable resource for further studies. The LiSEQ isolates have all been typed using current molecular methods and, thus, can be used to demonstrate the back compatibility of WGS with historical data and also to assess bioinformatic programmes that are able to predict such typing results from WGS data. WGS has allowed us to define the population of *L. monocytogenes* from this study to an unprecedented resolution. It has provided the framework to answer questions on genetic diversity amongst different sources assayed in this strain collection, as well as to explore possible epidemiological links between isolates.

Another application of WGS data is related to the improvement of quantitative microbial risk assessment [43, 44]. It has been recently proposed that more targeted risk assessments focused on subpopulations that pose the greatest risk should be performed, e.g. those that have an enhanced ability to survive or grow in the food chain or those considered to be more pathogenic [44–46]. The characterization of CCs, and virulence, stress and antibiotic markers of strains circulating in the EU in RTE foods, as described in this study, provides the opportunity for improved risk assessments for *L. monocytogenes* exposure [2].
