# Supplementary material for: LiSEQ – whole-genome sequencing of a cross-sectional survey of Listeria monocytogenes in ready-to-eat foods and human clinical cases in Europe
Source: Microb Genom. 2019 Feb 18;5(2):e000257. doi: 10.1099/mgen.0.000257 (PMC6421348; doi:10.1099/mgen.0.000257)
Supplement: Supplementary File 1 [file mgen-5-257-s001.pdf]

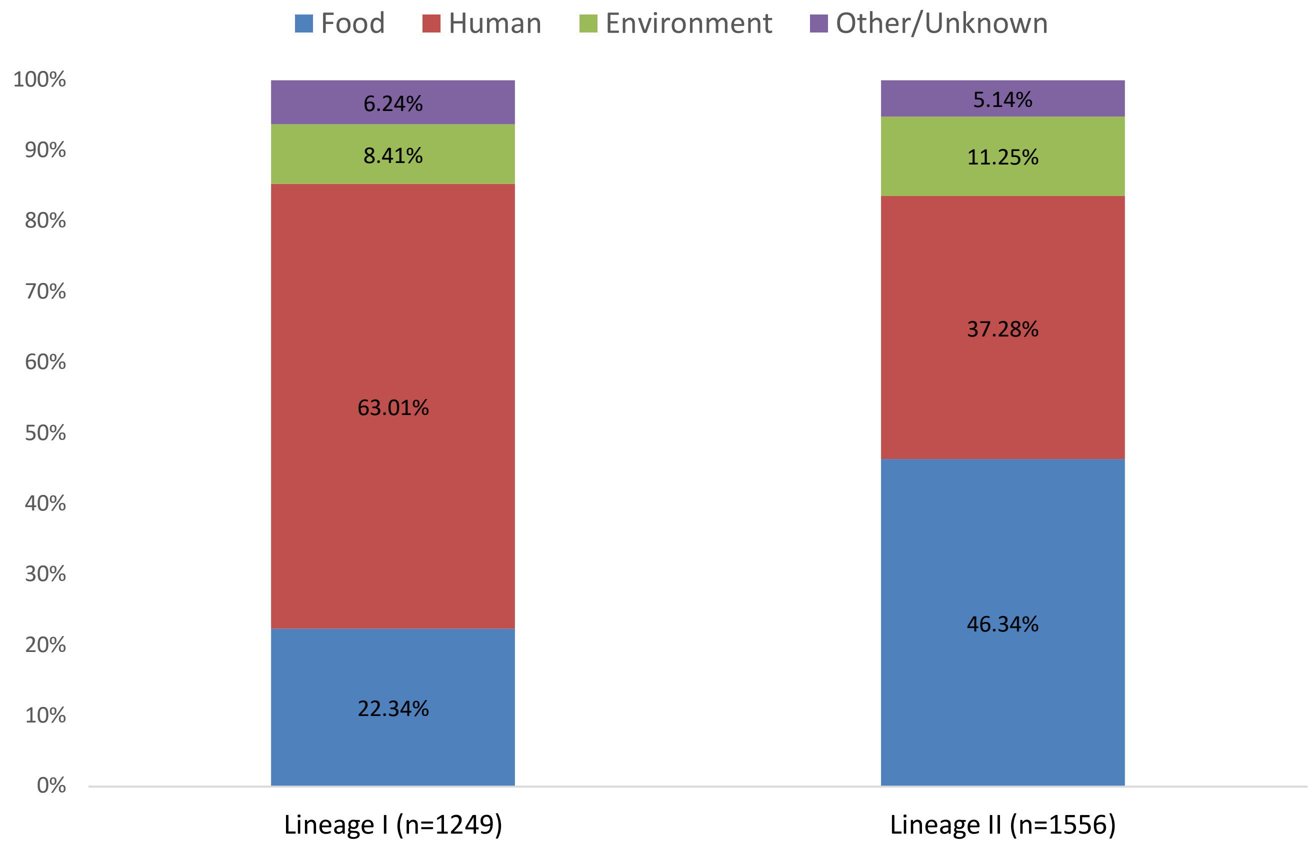

Supplementary Figure 1. Source of the isolates per lineage in the combined LiSEQ / Moura *et al,*. (2016) datasets

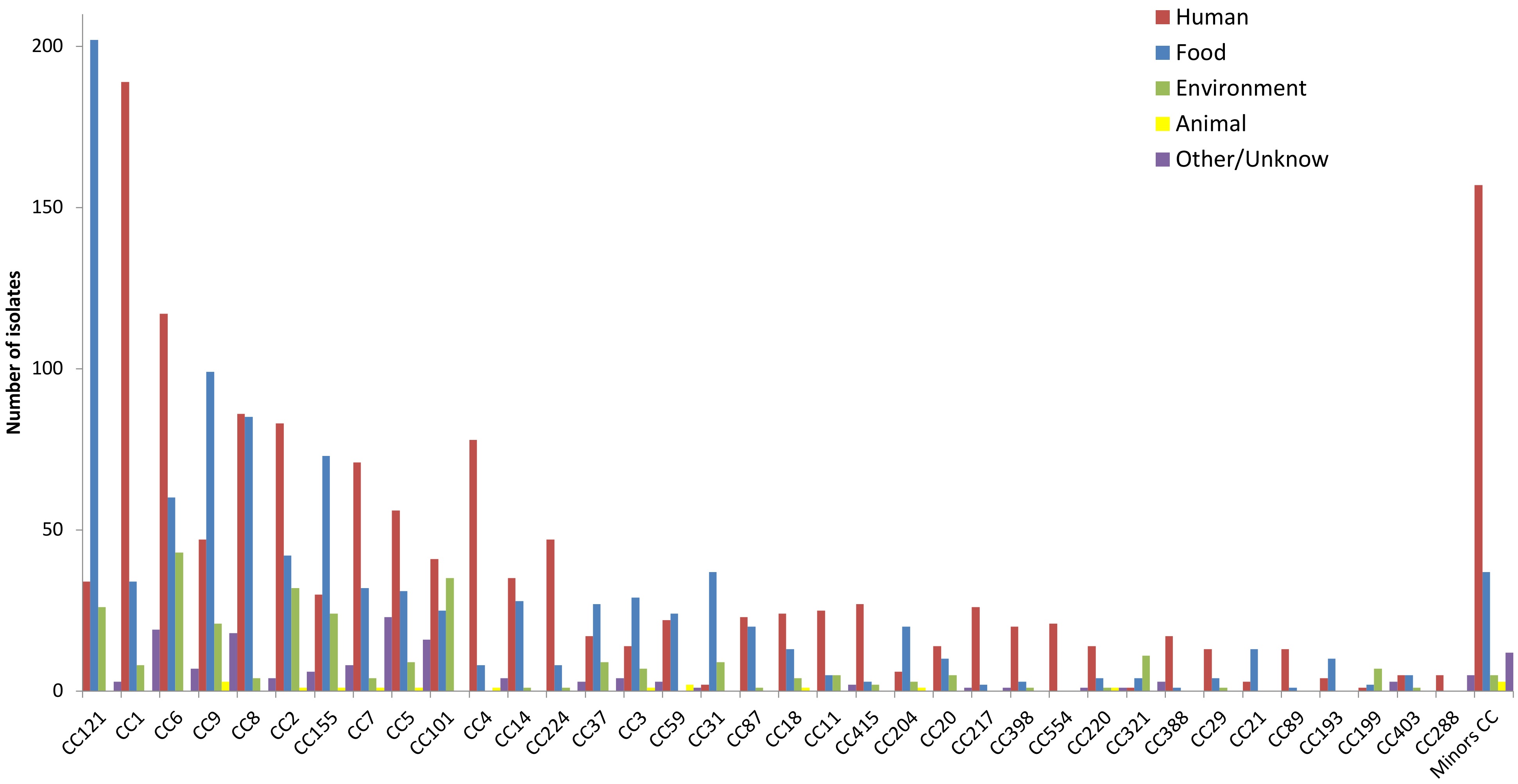

Supplementary Figure 2. Source of the isolates per clonal complex in the combined LiSEQ / Moura *et al* (2016) datasets
